# MRI of Implantation Sites Using Parallel Transmission of an Optimized Radiofrequency Excitation Vector

**DOI:** 10.3390/tomography9020049

**Published:** 2023-03-08

**Authors:** Mostafa Berangi, Andre Kuehne, Helmar Waiczies, Thoralf Niendorf

**Affiliations:** 1 Berlin Ultrahigh Field Facility, Max-Delbrück-Center for Molecular Medicine in the Helmholtz Association, 13125 Berlin, Germany; 2 Charité—Universitätsmedizin Berlin, 10117 Berlin, Germany; 3 MRI.TOOLS GmbH, 13125 Berlin, Germany

**Keywords:** MRI, implant, parallel transmission, transmission field shimming, safety, radiofrequency, RF array

## Abstract

Postoperative care of orthopedic implants is aided by imaging to assess the healing process and the implant status. MRI of implantation sites might be compromised by radiofrequency (RF) heating and RF transmission field ($B_{1}^{+}$) inhomogeneities induced by electrically conducting implants. This study examines the applicability of safe and $B_{1}^{+}$-distortion-free MRI of implantation sites using optimized parallel RF field transmission (pTx) based on a multi-objective genetic algorithm (GA). Electromagnetic field simulations were performed for eight eight-channel RF array configurations (*f* = 297.2 MHz), and the most efficient array was manufactured for phantom experiments at 7.0 T. Circular polarization (CP) and orthogonal projection (OP) algorithms were applied for benchmarking the GA-based shimming. $B_{1}^{+}$ mapping and MR thermometry and imaging were performed using phantoms mimicking muscle containing conductive implants. The local SAR10g of the entire phantom in GA was 12% and 43.8% less than the CP and OP, respectively. Experimental temperature mapping using the CP yielded ΔT = 2.5–3.0 K, whereas the GA induced no extra heating. GA-based shimming eliminated $B_{1}^{+}$ artefacts at implantation sites and enabled uniform gradient-echo MRI. To conclude, parallel RF transmission with GA-based excitation vectors provides a technical foundation en route to safe and $B_{1}^{+}$-distortion-free MRI of implantation sites.

## 1. Introduction

Postoperative care of orthopedic implants is aided by the imaging of the implantation sites to monitor the healing process of surrounding bone and tissues and to assess the implant status [1,2]. For this purpose, X-ray-based imaging modalities (e.g., CT, and radiography) are commonly applied in clinical practice [3]. The use of ionizing radiation in the longitudinal examination of implantation sites adds to the cumulative radiation dose of patients, which may be associated with an increased risk of malignancy [4].

MRI presents a viable alternative for the examination of implantation sites [5,6,7,8]. The metallic and electrically conductive nature of implants constitutes a challenge for MRI [9,10]. Metallic implants may induce magnetic susceptibility dispersions at implant–tissue interfaces [11,12]. The resulting magnetic field perturbations may compromise the anatomic integrity of MRI due to distortions, may induce signal loss due to shortening of the effective transversal relaxation time T_2_* or even signal voids in areas with very high B_0_ gradients, or may cause off-resonance effects caused by ΔB_0_-induced frequency dispersions [11]. These constraints can be addressed using on- and off-resonance approaches that permit MRI of implants free of B_0_ distortions and signal voids [13,14,15,16,17].

Another constraint of MRI monitoring of implantation sites is related to the interactions between conductive implants and radio frequency (RF) electromagnetic fields (EMF). These interactions lead to scattered EMFs originating from the implant [9]. The superposition of incident E-fields (${\vec{E}}_{inc}$) and scattered E-fields $\left({\vec{E}}_{sca}\right)$ may lead to locally elevated total E-fields: $\left|{\vec{E}}_{total}\right|=\left|{\vec{E}}_{inc}+{\vec{E}}_{sca}\right|$. This increases the specific absorption rate (SAR) by $\mathrm{SAR}\propto |{\vec{E}}_{total}{|}^{2}$ and may cause RF-induced heating [18]. For example, at 7.0 T MRI the RF wavelength (λ) in brain tissue is sufficiently short (λ~12 cm) to allow for resonance and heating effects at λ/4–λ/2, which is in the size range of clinically available orthopedic implants.

The magnetic component of scattered and incident superposition fields may induce RF transmission field $\left(B_{1}^{+}\right)$ inhomogeneities. These interferences may manifest as non-uniform image intensities, signal shading, signal voids, or signal intensity elevation in the vicinity of the implant, all of which bear the potential to spoil the benefits of MRI due to non-diagnostic image quality. Owing to the shape, location, and orientation of a conductive implant, also depending on the RF excitation vector in parallel RF transmission (pTx), the level of RF-induced heating and RF transmission field distortions may vary [18,19].

A plethora of reports present MRI hardware and methodology tailored for the examination and mitigation of RF-induced implant heating and/or of RF shading near conductive implants [20,21,22]. A reduction in RF heating at the tip of metallic implant leads and the improvement of $B_{1}^{+}$ homogeneity have been demonstrated by changing the magnitude of the excitation currents on two separate channels of a dual-drive birdcage RF coil [23]. Utilizing pTx pulse design at 3.0 T to reduce SAR near a deep brain stimulation device (DBS) in a uniform flip-angle excitation scheme has been implemented and verified in simulations [24]. The impact of RF coil configurations using pTx at 3.0 T has been investigated in numerical simulations, in phantom studies, and in cadaver studies with the goal of reducing the absorbed power or of improving transmission field uniformity around DBS implants [25,26]. An optimization procedure based on a subject-dependent optimization factor has been proposed to limit SAR while providing uniform $B_{1}^{+}$ [27]. A more general mathematical approach has been proposed for implant-friendly MRI and was previously demonstrated in a theoretical cylindrical model [28]. However, directly translating this approach to an actual coil array is not feasible. Firstly, the desired B_1_ profile and zero E-field in the implant were implemented as strict constraints in the optimization formulation. Secondly, the degrees of freedom afforded by a realistic pTx coil array are limited by the number of transmit channels. These two restrictions taken together result in a potentially unsolvable optimization problem because the constraints cannot be all simultaneously satisfied by the limited number of transmit channels. Other pioneering approaches include modification of RF transmission fields using RF arrays and parallel transmission with maximum- and null-current modes [29]. Machine-learning-based prediction of RF power absorption or ultrafast calculation of RF field enhancements near medical implants provide computational solutions for implant-specific RF heating assessment and management [30,31].

Considering the increasing population of patients with orthopedics implants [32], understanding and managing the interactions of conductive implants with RF fields is of profound importance for advancing safe and $B_{1}^{+}$-distortion-free MRI of implantation sites. This need concerns conventional titanium or stainless-steel-based implants and clinically available Mg-based biodegradable implants [33,34] which promote patient comfort and reduce healthcare costs by making implant removal surgery obsolete. This involves particularly small biodegradable screw or fixation implants (short implant), which are used as a real-clinical-world example in our study.

Recognizing the need for safeguarding MR monitoring of implants, this study examines the feasibility of moving towards safe and $B_{1}^{+}$-distortion-free MRI of implantation sites using parallel RF transmission. The main goal of our strategy is to exploit the degrees of freedom of multi-channel RF transmission using an optimized excitation vector that offsets the interactions between RF fields and a metallic implant. Our study adds to the literature because the objective of our approach of tailoring the total superposition of the RF fields is twofold: (1) to mitigate implant tip heating while keeping (local) SAR everywhere within the safety limits [35] and (2) to ensure $B_{1}^{+}$ homogeneity and uniform image quality in close vicinity of an implant. For this purpose, multi-channel RF array configurations comprising loop and dipole RF elements were designed and customized for 7.0 T MRI (*f* = 297.2 MHz) and examined in numerical EMF simulations to detail the $B_{1}^{+}$ E-fields and local specific absorption rates. The excitation vectors used for multi-channel transmission were derived from a multi-objective, genetic-algorithm-based optimization which demonstrates the novelty of our work. To advance from EMF simulations to a realistic clinical setup, the most efficient RF array configuration was manufactured, and its performance was assessed in phantom studies. To achieve this goal, RF transmission field mapping, MRI thermometry, and conventional MRI were performed using tissue-mimicking phantoms containing conductive implants.

## 2. Materials and Methods

The methods and materials used in this study are outlined in four sections:

1. The RF transceiver array configurations section introduces the design aspects and EMF simulations conducted to identify an optimum RF array configuration based on commonly used RF transceiver elements.
2. The transmission field shaping ($B_{1}^{+}$ shimming) section outlines the excitation vector optimization to minimize scattered fields.
3. The phantom experiments section details the setup used for validation of the EMF simulations in phantom studies conducted at 7.0 T.
4. The sections on MR thermometry and transmission field mapping describe the metrics used for validation.

### 2.1. RF Transceiver Array Configurations

**Figure 1:**
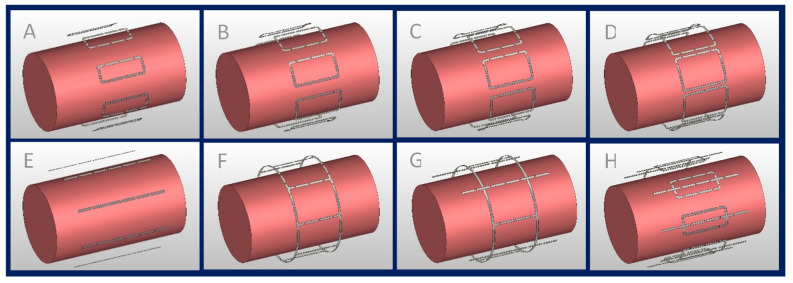
Eight-channel RF transceiver array configurations examined in the EMF simulations; (**A**–**D**) eight loop elements with L = 100 mm and W ≈ 41 mm, 52 mm, 62 mm, and 72 mm, (**E**) eight fractionated dipoles (L = 200 mm), (**F**) degenerate birdcage with eight rungs (L = 100 mm, D = 210 mm), (**G**) hybrid birdcage with four rungs (L = 100 mm, D = 210 mm) and four dipoles (L = 200 mm), and (**H**) loop–dipole array (L_loop = 100 mm, W_loop ≈ 41 mm, L_dipole = 200 mm).

To investigate the interference between electrically conductive implants, E-fields, and B-fields, numerical EMF simulations were performed at 297.2 MHz (operating frequency at 7.0 T MRI). A set of eight RF array configurations (Figure 1) tailored for MRI of body extremities comprising loops and/or fractionated dipoles [36] were evaluated aiming to identify the configuration with the best $B_{1}^{+}$ and SAR performance:

(A–D) Eight identical loop elements (L = 100 mm) with different widths (W), defined by $W_{n}=\left(\left(210\;\mathrm{mm}\right)\pi /16\right)\times \alpha_{n}$, where $n=1–4\;\mathrm{and}\;\alpha_{n}=1\;,\;1.25,\;1.5\;\mathrm{and}\;1.75$.

(E) Eight fractionated dipoles (L = 200 mm, W = 5 mm).

(F) Degenerate birdcage RF resonator (L = 100 mm, D = 210 mm) using eight rungs.

(G) Combination of a degenerate birdcage RF resonator (L = 100 mm, D = 210 mm, four rungs) and four fractionated dipoles (L = 200 mm, W = 5 mm), with a fractioned dipole being placed between each birdcage rung.

(H) Eight modules consisting of a loop (L = 100 mm, $W=\left(210\;\mathrm{mm}\right)\pi /16$) and a fractionated dipole [37,38] (L = 200 mm, W = 5 mm) placed in the center of the loop.

All RF transceivers were placed equidistantly around a cylindrical phantom (L = 300 mm, D = 170 mm) mimicking the electrical properties of muscle tissue $\varepsilon_{r}=58.24,\;\sigma =0.769\;(S/m$) at 297.2 MHz. The RF transceiver arrays were placed 20 mm away from the phantom and shielded at 30 mm.

The RF array configurations were implemented in CST Studio Suite 2020 (CST MWS, Darmstadt, Germany) using the Finite Integration Technique (FIT) [39]. EMF simulations were performed with smaller than 1.5 mm^3^ mesh resolution. Matching and tuning capacitors were set to force the magnitude of scattering parameters (both reflections and transmissions) of the system to less than −15 dB. Neighboring loop elements were decoupled with transformers [40]. Due to the geometric distance, no decoupling was required for the dipole elements. A cylindrical element (L = 70 mm, R = 1 mm,$\;\sigma =5.8\times 10^{8}\;S/m$) mimicking a conducting implant was placed inside the phantom at a depth of 30 mm from the surface parallel to the phantom axis. This simulation setup was used to assess the performance of the RF arrays in terms of strength and uniformity of $B_{1}^{+}$ in a cylindrical ROI (L = 110 mm, R = 20 mm) covering the implant, as well as the maximum induced SAR (averaged over 10 g tissue, SAR_10g,max_) in the entire phantom.

### 2.2. Transmission field Shaping ($B_{1}^{+}$ Shimming)

Transmission field shaping was performed to obtain a set of excitation vectors that met the requirements of (i) achieving a strong and uniform $B_{1}^{+}$ in the target ROI containing the implant and (ii) reducing the maximum local SAR below the limits imposed by the IEC guidelines [35]. This was achieved using the MATLAB (The Mathworks, Natick, MA, USA) toolbox [41] for multi-objective genetic algorithm (GA). The GA-based approach provides solutions for optimization problems with several conflicting objectives. For the field shaping problem, the output of the optimization is a set of excitation vectors that best satisfies the conflicting objectives. This set of solutions lies on a trade-off curve (pareto front) which illustrates the conflict between objectives, i.e., improving one objective results in the worsening of one or more other objectives. The following parameters were used for the definition of the objectives:

- ${\mathrm{local}\;\mathrm{SAR}}_{10g,\max}$: the maximum 10 g SAR value in the entire phantom, not just the implantation site.
- $B_{1}\_{\mathrm{SAR}}_{\max}=B_{1}^{+}/\sqrt{{\mathrm{local}\;\mathrm{SAR}}_{10g,\max}}$
- COV($B_{1}\_{\mathrm{SAR}}_{\max}$)$=\mathrm{std}\left(B_{1}\_{\mathrm{SAR}}_{\max}\;\right)/\mathrm{mean}\left(B_{1}\_{\mathrm{SAR}}_{\max}\right)$

where $B_{1}^{+}$ values are calculated for the target ROI containing the implant. Mean($B_{1}\_{\mathrm{SAR}}_{\max}$) is responsible for regulation of the $B_{1}^{+}$ strength as well as reduction in local SAR through the entire phantom. The uniformity of $B_{1}^{+}$ is controlled by the coefficient of variation ($\mathrm{COV}\left(B_{1}\_{\mathrm{SAR}}_{\max}\right))$. The output of this optimization is a complex excitation vector, the GA excitation vector ($U_{GA}$):

$${\vec{U}}_{GA}=K_{GA}\times \left(u_{1},\;u_{2},\;\ldots ,\;u_{8}\right)\;\mathrm{and}\;\left|u_{n}\right|=1;1\leq n\leq 8,\;n\in \mathbb{N}$$

where $K_{GA}$ is a real value constant that controls the overall excitation vector power, and $u_{n}$ are complex excitation values corresponding to each RF channel.

The optimization tolerance function was set to 10^−6^ so that the algorithm remained sensitive to small SAR_10g,max_ variations. Also for the optimization step, the SAR matrices were compressed using the virtual observation point [42] (VOP) approach.

The performance of the GA excitation vector to provide a strong and uniform $B_{1}^{+}$ pattern was benchmarked against the circular polarization (CP) mode [43]. The CP mode is a commonly used excitation vector which corresponds to a simple “Birdcage”-mode excitation used as a reference. The CP-mode vector ($U_{CP}$) is defined as:

$${\vec{U}}_{CP}=K_{CP}\times \left(u_{1},\;u_{2},\;\ldots ,\;u_{8}\right),\;u_{n}=\exp\left(-2\pi i\cdot n/8\right);\;1\leq n\leq 8,\;n\in \mathbb{N}.$$

where $K_{CP}$ is a real value constant to control the overall excitation vector power, i is the imaginary unit, and $u_{n}$ are complex excitation values corresponding to each RF channel.

The performance of the GA to reduce SAR_10g_ induced by the implant was benchmarked against the orthogonal projection (OP) method [22]. In the OP method, the implant-induced SAR is eliminated by projecting $U_{CP}$ (or any other excitation vector) onto a vector perpendicular to the vector creating the worst-case implant SAR $\left(U_{wc}\right)$. With this approach, the OP method supports elimination of the implant-induced SAR while the overall transmission field uniformity benefits from the advantages of the $U_{CP}$ excitation.

$${\vec{U}}_{OP}={\hat{U}}_{CP}-{\hat{U}}_{wc}\;\left({\hat{U}}_{CP}\;.\;{\hat{U}}_{wc}\right),$$

$${\hat{U}}_{CP}=\frac{{\vec{U}}_{CP}}{\left\|{\vec{U}}_{CP}\right\|},\;{\hat{U}}_{wc}=\frac{{\vec{U}}_{wc}}{\left\|{\vec{U}}_{wc}\right\|}.$$

The $U_{wc}$ is calculated as the eigenvector corresponding to the maximum eigenvalue of the local RF power correlation matrix in the target ROI [44].

### 2.3. Phantom Experiments

For validation of the EMF simulations, phantom experiments were performed at 7.0 T. Cylindrical phantoms (L = 300 mm and D = 170 mm) identical to those used in the EMF simulations were employed. A liquid-sucrose-based phantom [45] plus a solid polyvinylpyrrolidone (PVP)-based [46] phantom were used to emulate the electrical properties of muscle tissue at 297.2 MHz. The liquid-sucrose-based phantom was used for conventional MRI and $B_{1}^{+}$ mapping as it allows implant reorientation. Thermal experiments were conducted on the PVP phantom because there are no interfering ^1^H resonance peaks available in the NMR spectra of PVP which elevates the accuracy of MR thermal measurements [46].

To mimic the thermal behavior of biological tissue without additional fluid dynamics caused by thermal convection, a mixture of PVP $\left(33.9\%\;w/w\right)$, agarose $\left(0.4\%\;w/w\right)$, and NaCl (1.1% w/w) was dissolved in deionized water. For the sucrose-based phantom, no gel agent was used (sucrose 48.9%w/w, NaCl 1.9% w/w). A conductivity of σ = 0.77 S/m was used to match the conductivity of muscle tissue based on the electrical properties of various body tissues for a broad frequency range [47]. The permittivity was set to ε_r_ = 58.

**Figure 2:**
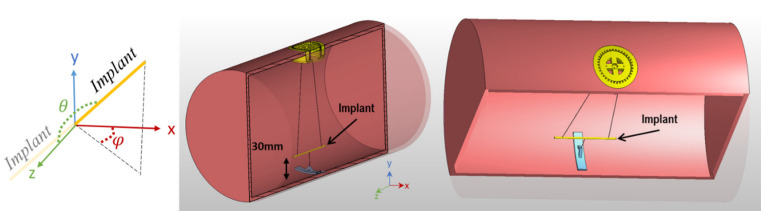
Cross section of the phantom and implant positioning system. The implant is suspended using cotton strings at a 30 mm distance from the phantom surface. The orientation of the implant can be controlled by adjusting the length of the strings and the rotation of the yellow implant adjuster positioned at the surface of the phantom. The orientations are defined based on a spherical coordinate system using polar (θ) and azimuthal (ϕ) angles when the origin is aligned with the center of the implant.

A copper wire (L = 70 mm, outer diameter D_out_ = 1 mm) mimicking an implant was placed inside the phantoms to emulate a conducting implant. The maximum implant length was chosen based on the maximum screw length of biodegradable implants commercially and clinically available today (www.syntellix.de, accessed on 1 March 2022). This approach provides a reasonable approximation of an implant because the induced current distribution on a metallic implant is the source of scattered fields which is less sensitive to the shape details [48] and metal characteristics [49]. Acrylic glass (PMMA) material was used as a phantom container. The implant was suspended in the phantom using cotton strings to minimize unwanted interference with EMFs. The strings were fixated with a 3D-printed setup made of Acrylonitrile Butadiene Styrene material to facilitate rapid and accurate positioning of the implant (Figure 2).

### 2.4. MR Thermometry

Implant-induced heating of GA- and CP-based excitation vectors was assessed by MR thermometry on the PVP phantom. MR thermometry was performed at the iso-center of the MRI scanner at room temperature (T = 297 K). Temperature difference maps were obtained using gradient-echo imaging (spatial resolution = 1.3 × 1.3 × 5.0 mm^3^, TE_1_ = 2.26 ms, TE_2_ = 6.34 ms, TR = 246 ms) in conjunction with the proton resonance frequency shift approach [50] before and after RF-induced heating. An additional oil sample was used within the field of view to compensate for the magnetic field drift [51]. For the RF heating paradigm (P_in_ = 175 W, duration = 5 min), a turbo-spin-echo technique was applied.

### 2.5. Transmission Field Mapping

The transmission field shimming methods were evaluated using low flip angle gradient echo imaging-based [52,53] $B_{1}^{+}$ mapping (TR = 10 s, TE = 2.31 ms, number of averages = 4, matrix size = 256 × 256, slice thickness = 5 mm) of transversal (FOV_transversal_ = 200 mm × 200 mm) and sagittal (FOV_sagittal_ = 250 mm × 250 mm) slices through the center of the implant which was aligned with the center of the phantom. This procedure was used for the worst-case scenario orientation, where the implant is aligned parallel to the main magnetic field B_0_ and parallel to the E-field lines of the RF arrays, thus ensuring maximum RF coupling between the E-field and the implant. The non-gel sucrose-based phantom, which enables convenient rotation of the implant, was used for $B_{1}^{+}$ mapping of a broad range of implant orientations.

Discrepancies in EMF patterns between the simulations and the experimental measurements may be due to losses or phase shifts which are introduced because of non-ideal real-world lumped elements, coupling of RF channels to the surroundings, and other factors. Small variations may accumulate and lead to a detectable effect on the RF field pattern. This is especially important in the close vicinity of the implant where the EMFs undergo significant alterations. Having exact information on the behavior of the EMFs in this region is important to suppress the implant-induced effects. These discrepancies were minimized in a calibration step including a simulated $B_{1}^{+}$ map ($B_{1,s}^{+})$ and its corresponding experimental map ($B_{1,e}^{+}$) for a slice close to the tip of the implant. This target slice can be selected in such a way that no interference from the implant is observed, or alternatively a slice-including implant can be selected if any invalid data in the implantation regions are masked. Then, complex calibrating coefficients were calculated to minimize the differences between measured and simulated $B_{1}^{+}$ maps in an optimization algorithm with the following error function:

$$\underset{X}{\min}\|\left(X*X_{e}*B_{1,s}^{+}\right)-\left(X_{e}*B_{1,e}^{+}\right)\|$$

where $B_{1,s}^{+}$ and $B_{1,\;e}^{+}$ are n×m complex matrixes. X is the optimization variable (calibration coefficients), and $X_{e}$ is the excitation vector used to acquire $B_{1,\;e}^{+}$. X and $X_{e}$ are 1×n complex vectors, n is the number of channels in the array, and m is the total number of pixels in $B_{1,\;e}^{+}$. These calibration coefficients are then multiplied by the excitation vectors obtained from the simulations to calculate the excitation vector used for the MRI experiments.

The efficacy of the GA shimming approach was investigated for several scenarios by changing the orientation of the implant. Different orientations were defined using a spherical coordinate system where the origin of the coordinate system is aligned with the center of the implant, and θ and φ are azimuthal and polar angles, respectively. The results obtained for the GA approach were benchmarked against the CP and the OP reference methods.

### 2.6. MR Hardware

The simulated values of the tuning and matching network were used as an initial starting point and adjusted to reach −15 dB for all scattering parameters (both reflections and transmissions) in the manufactured RF transceiver array. For phantom experiments, the RF transceiver was connected to a 7.0 T MRI scanner (Magnetom, Siemens, Erlangen, Germany) using a multi-channel interface (MRI.TOOLS GmbH, Berlin, Germany) containing transmit–receive switches and RF power dividers. The scanner was driven in pTx mode with precise control of the phase and amplitude for each of the eight RF channels.

## 3. Results

### 3.1. EMF Simulations of Eight-Channel RF Transceiver Configurations

**Figure 3:**
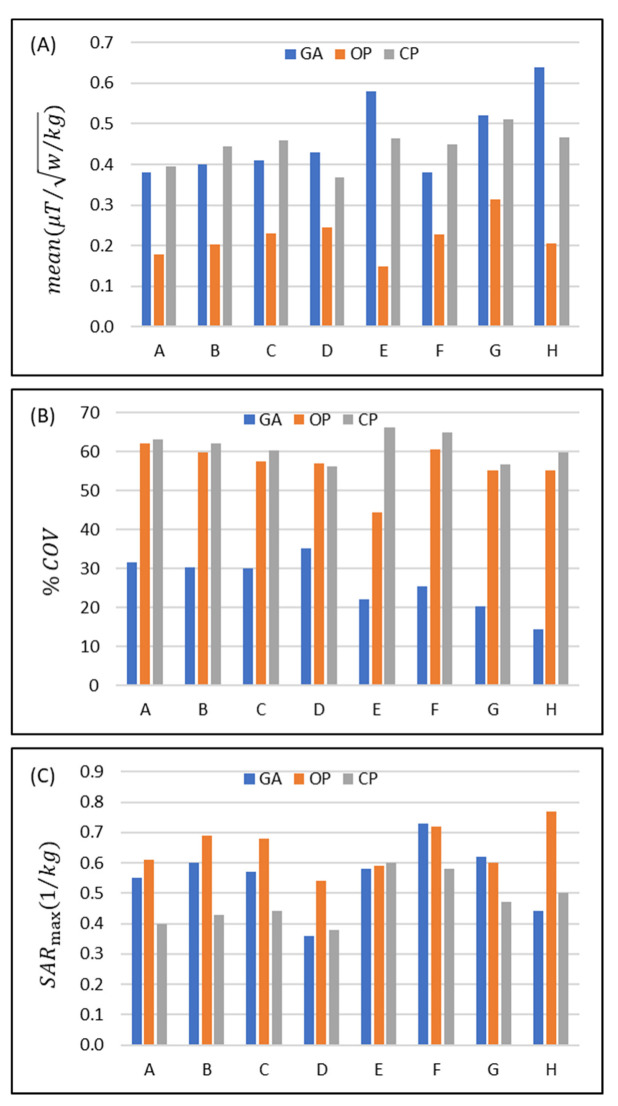
Comparison of the performance of the eight eight-channel RF transceiver configurations (design A to H) using a circular polarization (CP)-, an orthogonal projection (OP)-, and a multi-objective genetic algorithm (GA)-based approach for transmission field shimming. (**A**) Mean($B_{1}\_{\mathrm{SAR}}_{\max}$) and (**B**) $\;\%\mathrm{COV}\left(B_{1}\_{\mathrm{SAR}}_{\max}\right)$ in the ROI. (**C**) SAR_10g,max_ in the whole phantom.

The performance of the eight RF transceiver array configurations was assessed using the CP-mode, OP, and GA-derived transmission field shimming. The safety of each excitation is limited by the ${\mathrm{local}\;\mathrm{SAR}}_{\max}$. The strength and uniformity of each excitation vector in the target ROI was assessed by the mean($B_{1}\_{\mathrm{SAR}}_{\max}$) and coefficient of variation (COV($B_{1}\_{\mathrm{SAR}}_{\max}$)). The results obtained from the EMF simulations demonstrate that with GA-based shimming, the mean($B_{1}\_{\mathrm{SAR}}_{\max}$) is increased from configuration A to E and from F to H (Figure 3A). The mean($B_{1}\_{\mathrm{SAR}}_{\max}$) across all configurations for GA, CP, and OP is $0.42\pm 43\%\;\left(\mu T/\sqrt{w/\mathrm{kg}}\;\right),\;0.44\pm 9.4\%\;\left(\mu T/\sqrt{w/\mathrm{kg}}\;\right),\;\mathrm{and}\;0.22\pm 21\%\;\left(\mu T/\sqrt{w/\mathrm{kg}}\right)$, respectively.

Increasing the width of the loop elements used in configurations A-D increases mean($B_{1}\_{\mathrm{SAR}}_{\max}$) for each transmission field shimming algorithm (except for the CP mode in D) (Figure 3A). The mean($B_{1}\_{\mathrm{SAR}}_{\max}$) obtained for GA versus CP transmission field shimming (%(Mean_GA_/Mean_CP_ − 1) = −3.6% (A), −9.9% (B), −10.9% (C), and 16.8% (D)) and OP versus CP transmission field shimming (%(Mean_OP_/Mean_CP_ − 1) = −54.6% (A), −54.1% (B), −49.8% (C), and −33.2% (D)) reveals that the OP approach is inferior to the CP and GA approaches.

For the dipole-only configuration (E) and for the eight-loop–dipole (H) configuration, the mean $\left(B_{1}\_{\mathrm{SAR}}_{\max}\right)$ obtained from the GA outperforms the CP approach (%(Mean_GA_/Mean_CP_ − 1) = 25.3% and 37.3%) and is superior to the OP algorithm (%(Mean_OP_/Mean_GA_ − 1) = −74.5% and −67.8%).

For the degenerate birdcage array (F), the CP approach provided the largest mean($B_{1}\_{\mathrm{SAR}}_{\max}$) where (%(Mean_GA_/Mean_CP_ − 1) = −15.6%). In configuration (G), the mean($B_{1}\_{\mathrm{SAR}}_{\max}$) strength derived from GA yielded a small difference (%(Mean_GA_/Mean_CP_ − 1) = 1.6%) versus the CP algorithm, while the mean($B_{1}\_{\mathrm{SAR}}_{\max}$) obtained from the OP algorithm is much lower (%(Mean_OP_/Mean_CP_ − 1) = −38.7%).

Assessment of the $B_{1}\_{\mathrm{SAR}}_{\max}$ homogeneity revealed that the OP algorithm provided a transmission field uniformity similar to that obtained for the CP algorithm for all eight RF array configurations with the exception of configuration E (%(1 − COV_OP_/COV_CP_) = 1.6%, 3.9%, 4.6%, −1.4%, 32.8%, 2.8%, 6.6%, 7.5%) (Figure 3B). The GA provided a substantially more uniform transmission field pattern versus the OP or the CP approach (%(1 − COV_GA_/COV_CP_) = 49.7%, 51.3%, 50.1%, 37.2, 66.6%, 64.1%, 60.8%, 75.9%) (Figure 3B).

Assessment of the RF power deposition showed that average SAR_10g,max_ (1 W input power) was below 0.8 (W/kg) for all RF transceiver array configurations and RF transmission field shaping approaches (Figure 3C). For most of the eight RF transceiver configurations, CP provided a lower SAR than GA or OP. The SAR obtained for the GA approach was similar to that of the OP algorithm or less. The maximum SAR_10g,max_ derived from the GA approach for the loop–dipole (H) configuration was 12% and 43.8% less than the CP and OP counterparts.

The implant-induced hot SAR spots resulting from the CP mode were eliminated with the OP algorithm. Yet, our simulations showed that in some scenarios OP excitation vectors produced a superficial SAR_10g,max_ value that is outside of the implantation site, and thus its maximum SAR_10g,max_ is still in the range of the results derived from the CP mode.

Using the GA for transmission field shimming, the eight-loop–dipole configuration (H) yielded a 15.8% increase in SAR_10g,max_ versus the lowest SAR value among all configurations/shimming (available in configuration D with CP shimming). On the other hand, this increased SAR_10g,max_ is compensated for by providing the strongest mean($B_{1}\_{\mathrm{SAR}}_{\max}$) among all configurations/shimming (25% more than the second strongest mean($B_{1}\_{\mathrm{SAR}}_{\max}$); in configuration F with CP) and the most uniform $B_{1}\_{\mathrm{SAR}}_{\max}$ excitation pattern (COV% is 67.6% lower than the lowest COV% found for the OP algorithm in design G) in the target ROI containing the implant.

### 3.2. Phantom MR Experiments

**Figure 4:**
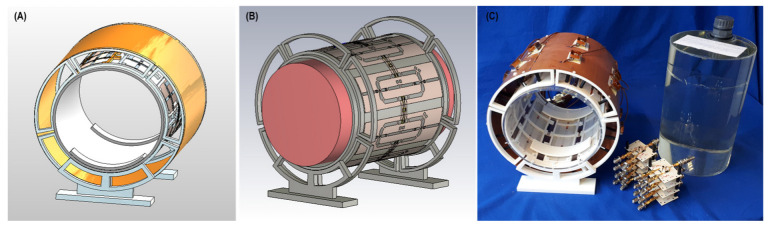
Experimental setup of the 8-channel loop–dipole RF transceiver array. (**A**,**B**) Computer-aided design of the RF transceiver array and the loop–dipole configuration decoupled with transformers. (**C**) Manufactured 8-channel loop–dipole RF transceiver array together with the phantom, the implant, the phantom container, and the power splitters used for RF feeding of the array.

Based on the EMF simulations, the eight-loop–dipole configuration (H) was selected for manufacturing an RF transceiver array for use in phantom experiments. The computer-aided design and a photo of the manufactured prototype of configuration H, along with the phantom container and power splitters used for feeding the RF array, are shown in Figure 4.

For this configuration, the efficiency of the GA-based shimming method versus the CP and OP approaches was examined using the PVP-based gel phantom. The implant was aligned with the long axis of the phantom as the E-field lines of the RF array are along this orientation, hence inducing the most implant SAR. The metrics investigated were $B_{1}^{+}$ $\left(\mu T/\sqrt{\mathrm{kW}}\right)$ strength and uniformity (mean and COV% of $B_{1}^{+}/\sqrt{P_{fwd.}}$, respectively, where $P_{fwd.}$ is the sum of the input power to all RF channels).

**Figure 5:**
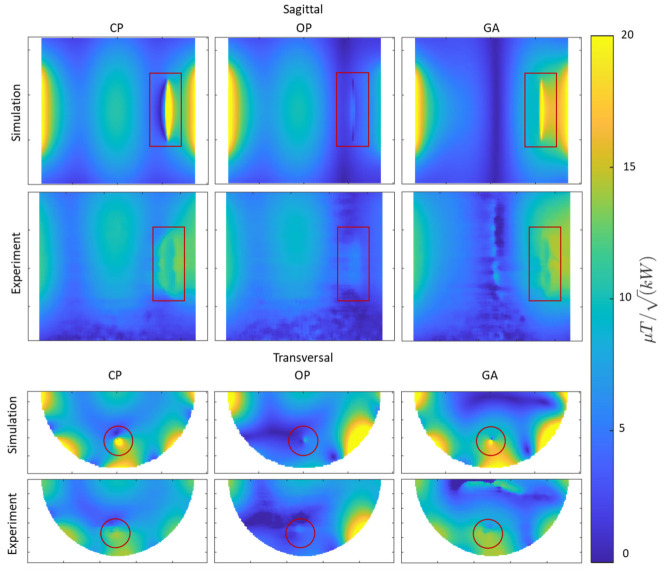
Simulated and experimental results obtained for $B_{1}^{+}$ mapping using CP-, OP-, and GA-based transmission field shimming algorithms. The top two rows show sagittal $B_{1}^{+}$ maps (covering the entire implant including tips). The bottom two rows show transversal $B_{1}^{+}$ maps (covering the regions with most pronounced RF distortion) derived from simulations and phantom experiments. The ROI containing the implant is indicated in red.

The experimental $B_{1}^{+}$ mapping results along with the corresponding $B_{1}^{+}$ maps obtained from the EMF simulations are shown in Figure 5 with the ROI containing the implant highlighted in red. The $B_{1}^{+}$ maps derived from the EMF simulations and the phantom experiments show good agreement. The simulated $B_{1}^{+}$ maps highlight that the CP approach suffers from a $B_{1}^{+}$ asymmetry around the implant, which manifests as a strong $B_{1}^{+}$ void on one side of the implant and a $B_{1}^{+}$ elevation on the opposite side. This asymmetry is reduced when using the OP algorithm. This improvement comes at the cost of $B_{1}^{+}$ destruction close to the implant. Unlike the CP and OP approaches, the transmission field vector obtained from the GA provides a uniform and increased $B_{1}^{+}$ field in the target ROI containing the implant.

**Figure 6:**
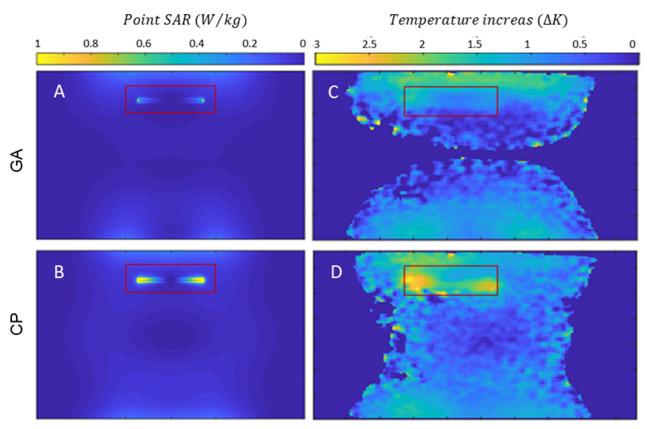
Simulation of point SAR maps (projection of maximum value) for a sagittal view of the phantom using GA- (**A**) and CP-based (**B**) transmission field shimming for 1 W input power along with temperature increase maps obtained for a sagittal view using GA- (**C**) and CP-based (**D**) shimming. The ROI containing the implant is indicated in red.

Next, the SAR reduction of the GA approach was investigated and benchmarked against the CP approach. Point SAR and temperature difference maps were derived from EMF simulations and from phantom experiments. The OP mode was not considered for heating evaluations due to its weak and non-uniform $B_{1}^{+}$ in the close vicinity of the implant. The point SAR distribution obtained from the EMF simulations shows a pattern similar to the E-field distribution, given that SAR is proportional to E^2^. When the implant was positioned parallel to B_0_, a dipole antenna effect was observed for SAR near the tips of the copper wire. This is due the accumulation of charges at the tips of the implant causing elevated SAR in the close vicinity of the implant. The movement of these charges on the surface of the implant (i.e., induced currents) is responsible for $B_{1}^{+}$ inhomogeneities. The SAR obtained for the GA-based transmission field shimming is substantially reduced compared to that of the CP mode, meaning that less current is induced on the implant with the GA approach (Figure 6). This SAR reduction is achieved by creating a reduced E-field in the vicinity of the implant.

Temperature difference maps (Figure 6) derived from MR thermometry confirmed the results obtained from the SAR assessment. The transmission field vectors obtained for the CP approach induced a temperature increase of ΔT = 2.5–3.0 K at the tips of the implant. The GA approach resulted in transmission fields which induced no extra temperature increase around the implant (Figure 6). With the GA approach, the area around the implant showed a temperature which did not differ from the background temperature distribution. A summary of the metrics obtained from the EMF simulations and the phantom experiments is shown in Table 1.

The simulated and measured $B_{1}^{+}$ maps and their corresponding point SAR and temperature difference maps obtained with the GA excitation vector demonstrated that a reduction in SAR is related to the homogenization of the $B_{1}^{+}$ field in the vicinity of the implant. This can be explained by the fact that both unwanted effects originate from the same source, namely induced currents on the conductive implant. Thus, the reduction in SAR is related to the homogenization of the $B_{1}^{+}$ field and vice versa.

**Figure 7:**
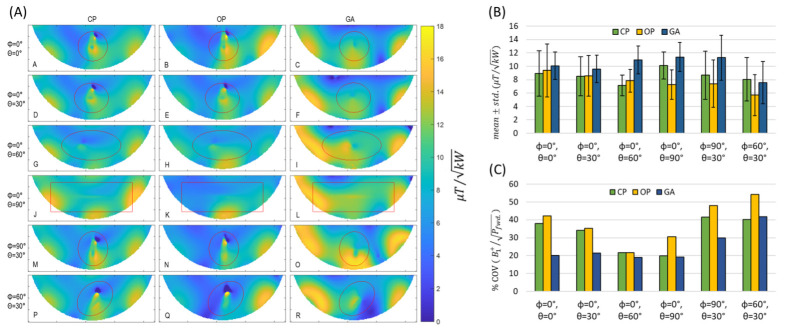
(**A**) Transversal experimental $B_{1}^{+}$ maps obtained for the tissue-mimicking phantom using CP-, OP-, and GA-based transmission field shimming for several implant orientations (Labelled from A to R). The implant orientation is defined using polar (θ) and azimuthal (φ) angles in spherical coordinates by considering the origin of the coordinate system on the center of the implant. The ROI covering the phantom is indicated in red. (**B**) Mean ± std. and (**C**) % coefficient of variation in $\;\left(B_{1}^{+}\right)/\sqrt{power_{Fwd.}}$ inside the ROI for different implant orientations (defined with polar (θ) and azimuthal (φ) angles with the origin of the coordinate system being placed on the center of the implant) using CP, OP, and GA transmission field shimming.

Next, the orientation of the implant was varied, and the $B_{1}^{+}$ maps measured, relative to the reference position (the implant was aligned with the long axis of the phantom). For convenient repositioning of the implant, the liquid-sucrose-based phantom was used. The implant orientations were defined using spherical coordinates where the origin was placed at the center of the implant, and polar (θ) and azimuthal (φ) angles defined the orientation. The transversal maps were acquired for slices through the center of the implant, where the implant-induced inhomogeneity of $B_{1}^{+}$ reaches a maximum. A summary of the $B_{1}^{+}$ maps obtained for the CP-, OP-, and GA-based shimming algorithms is shown in Figure 7A(A–R). For an orientation of θ=90° and φ=0°, the implant-induced $B_{1}^{+}$ artefact reached a minimum (Figure 7A(J–L)). For this orientation, a minimal current is induced on the implant because the E-Fields of the RF transceiver array are almost parallel to the long axis of the RF transceiver. Other implant orientations revealed strong $B_{1}^{+}$ inhomogeneities in the vicinity of the implant for transmission field shimming using the CP or OP algorithm. The GA supported substantial improvements in the $B_{1}^{+}$ uniformity. For every implant orientation, GA transmission field shimming provided a combined mean $B_{1}^{+}$ and $B_{1}^{+}$ uniformity which was superior to the counterparts derived from CP and OP transmission field shimming.

### 3.3. MRI of Implants Using a High Spatial Resolution

**Figure 8:**
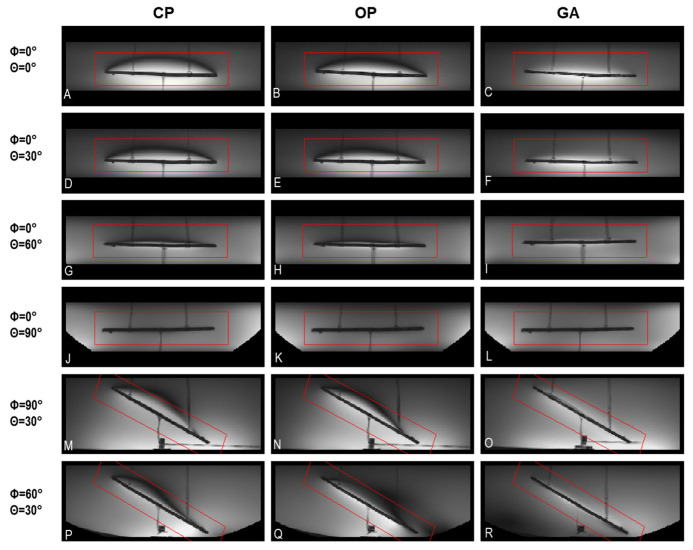
Minimum-intensity projections of worst-case $B_{1}^{+}$ artifacts obtained from 3D gradient-echo MRI in the vicinity of the implant using CP-, OP-, and GA-based transmission field shimming; (**A**–**R**) correspond to the excitation vector shown in Figure 7 for several implant orientations. The implant orientation is defined by using polar and azimuthal angles in spherical coordinates by considering the origin of the coordinate system on the center of the implant. The ROI under investigation is indicated in red.

To examine the clinical applicability of transmission field shimming, a 3D gradient-echo MRI was performed (TR = 20 ms, TE = 2.7 ms, FA = 20°, isotropic spatial resolution = 0.5 mm^3^, matrix size = 512 × 512 × 104, TA ≈ 17 min, receiver bandwidth = 501 Hz/Px) using the eight-loop–dipole configuration (H) in conjunction with the excitation vectors derived from the CP, OP, and GA approaches. From the 3D data sets, imaging planes including the implant and $B_{1}^{+}$ artifacts were manually selected using a custom-built MATLAB script. Minimum-intensity projection (MinIP) was used to project the 3D data in the vicinity of the implant onto 2D MinIP images (Figure 8) which help elucidate any destructive interference. For transmission field shimming using the CP and OP algorithms, a bow-shaped $B_{1}^{+}$ artefact is formed close to the implant. GA transmission field shimming eliminated $B_{1}^{+}$ artefacts and facilitated the acquisition of uniform images in the vicinity of the implant (Figure 8).

### 3.4. Simulations in the Realistic Human Voxel Model

**Figure 9:**
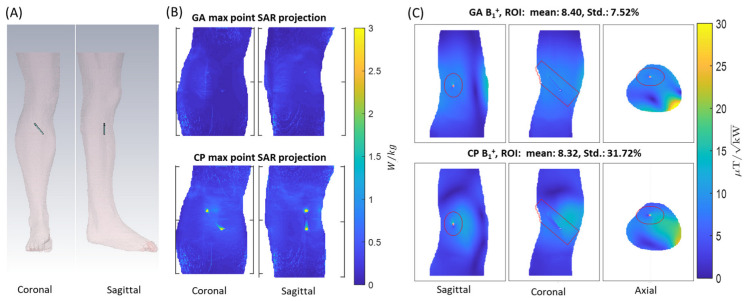
(**A**) Overview of the setup used for EMF simulations showing the positioning of the sample screw implant in the tibia of the human voxel model Duke. (**B**) Maximum point SAR projections obtained for the GA (**top**) and CP (**bottom**) approach for 1 W input power. (**C**) $B_{1}^{+}$ maps derived for slices through the center of the implant using the GA (**top**) and CP (**bottom**) approach. The ROI is highlighted in red.

The eight-loop–dipole configuration (H) was selected for the simulation of a realistic human model (Duke [54]) with a sample screw (L = 70 mm, outer diameter D_out_ = 1 mm) implanted in the right tibia. The GA with the properties described in the section on transmission field shaping ($B_{1}^{+}$ shimming) was implemented for a cylindrical ROI (L = 110 mm, D = 40 mm). The CP was used for benchmarking in terms of $B_{1}^{+}$ strength and uniformity in the ROI and SAR reduction, and the results are presented in Figure 9.

## 4. Discussion

MRI monitoring of tissue healing and implant status may be compromised by RF-induced tissue heating and transmission field inhomogeneities. Here, we demonstrate the feasibility of moving towards safe and $B_{1}^{+}$-distortion-free MRI at 7.0 T in the presence of implants, using parallel radiofrequency transmission in conjunction with excitation vector optimization. Eight RF array configurations comprising loop elements and/or fractionated dipoles were characterized in EMF simulations using the metrics SAR_10g,max_ and transmission field strength and uniformity. The EMF simulations demonstrated that the eight-channel loop–dipole RF array configuration driven with optimum transmission field patterns obtained from a multi-objective GA provided the strongest transmission field $B_{1}^{+}$ and the most uniform $B_{1}^{+}$ distribution for a target ROI containing the implant. $B_{1}^{+}$ mapping, MR thermometry, and 3D gradient-echo imaging of a phantom mimicking muscle tissue showed that parallel transmission using the eight-channel loop–dipole RF array in conjunction with the multi-objective GA successfully reduces implant-induced SAR and provides transmission field uniformity required for MRI-based monitoring of tissue healing and for monitoring the degradation state of metallic implants. While our feasibility study was performed at 7.0 T, this approach can be readily applied to any available pTx system at various magnetic field strengths of 3.0 T and 1.5 T. It is also suitable for higher magnetic field strengths such as 10.5 T or 14.0 T. Using dynamic pTx versus static pTx would permit further transmission field enhancement in the presence of implants. While our clinical example used for demonstration of proof of principle focuses on screw implants used for fixation of bone fractures in body extremities, our approach can be conveniently applied to other body regions including the use of RF arrays customized for these body regions.

The CP excitation approach results in $B_{1}^{+}$ artifacts and excessive implant-induced SAR close to the implant. The OP method can reduce implant-induced SAR, but only at the cost of $B_{1}^{+}$ degradation at the implant site, resulting in non-uniform image intensity. Our results demonstrate that the GA-based approach addresses both these challenges, and thus represents a promising option en route to safe clinical MRI of orthopedic implants, free of $B_{1}^{+}$ artifacts. It is a recognized limitation of our study that MRI was limited to high-spatial-resolution gradient-echo imaging. Further research into other MRI techniques such as echo-planar imaging or fast-spin-echo imaging is warranted.

GA-based transmission field shimming eliminates conducting implant effects on EMFs by the suppression of RF-induced current on the implant surface. This is achieved by creating a reduced E-field region in the implantation location. Hence, it is plausible that the GA method can be adapted for shaping the transmission field around other passively conducting (interventional) devices. These include, for example, standard titanium implants, catheters, intracoronary stents, guide wires, or metallic needles. This approach is compatible with the rapid detection and mitigation of RF-induced implant heating during MRI using small (<1.5 mm^3^) and low-cost (EUR < 1) root-mean-square (RMS) sensors, such as diodes and thermistors integrated within an implant [55]. Although current commercially available diodes and thermistor configurations are not yet biodegradable, continuing advances in bioderived materials, green processing, and additive manufacturing for green electronics offer a conceptually appealing strategy to pursue the development of biocompatible and biodegradable electronic devices, which can complement biodegradable orthopedic implants, allowing even more effective non-invasive monitoring [56,57].

A caveat of this feasibility study is that the number of RF transmission channels is limited to eight independent radiofrequency power amplifiers (RFPA, each 1 kW peak output power) due to the MR scanner system design used. However, recent commercially available implementations that support up to sixteen RFPAs, each providing up to 2 kW adjustable RF output power, can circumvent this limitation. Pioneering scalable prototypes supporting up to thirty-two independent signal generators, RFPAs, and RF chains suitable for parallel transmission MRI of the body at 7.0 T offer even more potential [58,59]. Parallel transmission with RF transceiver array configurations of up to 48 channels have also been evaluated in EMF simulations [60]. Thus, increasing the number of RF transmission channels will improve the degrees of freedom and will provide more flexibility for transmission field shaping. This advancement will be greatly beneficial for the suppression of induced currents on implants of arbitrary geometry or size and can potentially improve the overall performance of the approach proposed herein. Increasing the number of RF channels to cover the same region of interest requires smaller transceiver elements (due to limited space) which reduces load noise seen from each element but also introduces extra coil resistance (i.e., through more copper, lumped elements, etc.) to the total resistance seen from the RF transceiver ports [61] which constrains the signal-to-noise ratio of MRI. On the other hand, increasing the number of channels elevates the total losses in the transmission path as more cabling and circuit elements are required. Therefore, the ideal number of independent RF transmission channels used for MRI of implants will depend on the specific application, implant configuration, and target anatomy.

## 5. Conclusions

This study demonstrates that parallel transmission using an eight-channel loop–dipole RF array in conjunction with a multi-objective genetic algorithm for transmission field shaping ensures MR safety and transmission field uniformity suitable for MRI-aided monitoring of tissue healing of implantation sites including MRI characterization of the degradation state of biodegradable orthopedic implants. The proposed approach provides important guidance for RF coil design and provides a technological basis for MRI of orthopedic and other conducting implants at clinical magnetic field strengths. While the impact of the RF transmit array on the efficiency of GA-based excitation vector optimization is acknowledged, it stands to reason that the approach evaluated and validated in this study is compatible with any RF array with an arbitrary number of transmit channels to facilitate safe and $B_{1}^{+}$-distortion-free MRI of implants.

## Figures and Tables

**Table 1:** Summary of statistical $B_{1}^{+}$ parameters and maximum temperature increase derived from numerical EMF simulations and phantom experiments using the CP, OP, and GA approaches for transmission field shaping.

Transmission Field Shaping Algorithm	EMF Simulation			Experiment		
	$mean\;(B_{1}^{+})$ $\left(\mu T/\sqrt{kW}\right)$	$\%\frac{std.\left(\;B_{1}^{+}\right)}{mean\left(\;B_{1}^{+}\right)}$	max. ΔK	$mean\;(B_{1}^{+})$ $\left(\mu T/\sqrt{kW}\right)$	$\%\frac{std.\left(\;B_{1}^{+}\right)}{mean\left(\;B_{1}^{+}\right)}$	max. ΔK
CP	7.6	51.4%	3.27 K	7.3	53%	3.15 K
OP	3.2	53.2%	-	2.3	69%	-
GA	10.3	23.2%	1.31 K	9.6	23%	1.23 K

## Data Availability

The code and data that support the findings of this study will be openly available in GitHub (https://github.com/BerangiMostafa/safe_MRI_of_implants, accessed on 1 April 2023) upon publication of the manuscript.
